# Anal Human Papillomavirus Infection Progression to Disease Among Men Who Have Sex With Men

**DOI:** 10.1093/cid/ciaf584

**Published:** 2025-10-29

**Authors:** Joel M Palefsky, Anna R Giuliano, Stephen E Goldstone, Brady Dubin, Alfred Saah, Alain Luxembourg, Christine Velicer, Joseph E Tota

**Affiliations:** Department of Medicine, University of California San Francisco, San Francisco, California, USA; Center for Immunization and Infection Research in Cancer (CIIRC), Moffitt Cancer Center, Tampa, Florida, USA; Department of Surgery, Icahn School of Medicine at Mount Sinai, New York, New York, USA; Merck Research Laboratories, Merck & Co., Inc., Rahway, New Jersey, USA; Global Medical and Scientific Affairs, Merck Research Laboratories, Merck & Co., Inc., Rahway, New Jersey, USA; Vaccine Clinical Development, Merck Research Laboratories, Merck & Co., Inc., Rahway, New Jersey, USA; Department of Epidemiology, Biostatistics and Research Decision Sciences, Merck Research Laboratories, Merck & Co., Inc., Rahway, New Jersey, USA; Department of Epidemiology, Biostatistics and Research Decision Sciences, Merck Research Laboratories, Merck & Co., Inc., Rahway, New Jersey, USA

**Keywords:** human papillomavirus, HPV vaccines, HPV infection, anal intraepithelial neoplasia, anal condyloma

## Abstract

**Background:**

Critical gaps exist in our understanding of the progression from anal human papillomavirus (HPV) infection to same-type HPV-associated anal disease in human immunodeficiency virus (HIV)-negative men who have sex with men (MSM). We conducted a post hoc analysis of a phase 3 randomized 4-valent HPV vaccine trial (NCT00090285) to assess the natural progression of anal HPV infections to associated anal lesions among MSM.

**Participants:**

A total of 602 HIV-negative MSM aged 16–27 years.

**Methods:**

HIV-negative MSM aged 16–27 years enrolled in the placebo arm from 18 countries were included. We estimated the distribution of 9-valent HPV (9vHPV) vaccine types in intra-anal lesions (anal condyloma and anal intraepithelial neoplasia [AIN] 1–3), proportions and rates of progression from incident-persistent (no associated anal disease at baseline) intra-anal HPV infections to the same-type HPV-associated anal lesion, and cumulative incidence over 30 months.

**Results:**

Predominant 9vHPV types detected were HPV6 (55.7%) and HPV11 (25.3%) in MSM with anal condyloma/AIN1, and HPV6 (30.5%), HPV16 (22.0%), and HPV11 (15.3%) in those with pooled AIN2/3. Progression from incident-persistent intra-anal 9vHPV infection to anal disease was driven primarily by HPV6, HPV11, and HPV16/18 infections for MSM with anal condyloma/AIN1 (63.0%, 85.7%, and 16.3%, respectively) and AIN2/3 (25.9%, 14.3%, and 32.4%, respectively). Cumulative incidence of anal condyloma/AIN1 and AIN2/3 in MSM with incident-persistent infection was 43.1% and 34.4%, respectively.

**Conclusions:**

A high proportion of unvaccinated HIV-negative MSM with a new intra-anal 9vHPV infection developed same-type HPV-associated anal disease. These findings support HPV vaccination of young HIV-negative MSM to prevent anal HPV infection and associated anal disease.

**Registration:**

ClinicalTrials.gov, NCT00090285.

## Teaser

- A high proportion of young, unvaccinated human immunodeficiency virus (HIV)-negative men who have sex with men (MSM) with a new intra-anal HPV infection develop same-type HPV-associated anal disease.
- HPV vaccination of young HIV-negative MSM can prevent anal HPV infection and same-type HPV-associated anal disease.

Human papillomavirus (HPV) infections cause most cases of anal cancer and anogenital warts [1–3]; global data suggest that all cases of anal squamous cell carcinoma are attributable to HPV infection [4]. Among men, anal cancer was most commonly attributed to high-risk HPV16 infection, detected in approximately 70%–80% of anal cancer cases [5], whereas most anal condyloma were attributed to low-risk HPV6/11 infections, detected in approximately 50%–60% of anal condyloma cases [6, 7]. In particular, the global prevalence of anal HPV infection and high-grade squamous intraepithelial lesions or worse (grade 2 or 3 anal intraepithelial neoplasia [AIN 2 or 3 or anal cancer) was high among men who have sex with men (MSM) or without human immunodeficiency virus (HIV) [8, 9].

The prevalence of anal infections with 9-valent HPV (9vHPV) vaccine types (42.6%) and high-grade anal lesions (11.3%) is high in HIV-negative MSM [8]. However, critical gaps exist in our understanding of the progression of persistent anal HPV infection to associated anal disease in HIV-negative MSM. It is also important to define the rate at which persistent HPV infections progress to disease. Therefore, we conducted a study to assess the natural progression of incident-persistent and prevalent-persistent anal HPV infections to same-type HPV-associated anal lesions among MSM aged 21–27 years who were HIV negative.

## METHODS

### Data Source

This was a post hoc analysis of data from young MSM enrolled in the placebo arm of a global, randomized controlled trial of the 4-valent HPV (4vHPV) vaccine, which was previously reported (V501-020 trial; NCT00090285) [10, 11]. MSM were enrolled from Australia, Brazil, Canada, Croatia, Germany, Spain, and the USA. Briefly, key inclusion criteria were MSM (aged 16–26 years) with 1–5 lifetime sex partners who engaged in either insertive or receptive anal intercourse or oral sex with another male partner within the past year; no prior diagnosis of HIV infection; no evidence of anogenital lesions suggesting non-HPV sexually transmitted disease, and no history of, or clinical evidence of, anogenital warts or dysplasia [10, 11]. MSM with HIV were excluded [11].

The V501-020 trial was conducted in accordance with Good Clinical Practice Guidelines and applicable country or local statutes and regulations regarding ethical committee review, informed consent, and the protection of the rights and welfare of human participants in biomedical research. Participants (or their legally authorized representatives) provided written informed consent at the start of the study.

### HPV DNA Sampling and Assessment

On day 1, month 7, and at 6-month intervals through follow-up, intra-anal swab specimens were obtained from MSM for cytologic analysis and HPV DNA typing. Participants were also sampled for HPV DNA at the penis, scrotum, and perineum at each study visit. All anal specimens were HPV-genotyped using multiplex polymerase chain reaction (PCR)-based assays [12, 13] to identify DNA for the 9 HPV types targeted by the 9vHPV vaccine (6/11/16/18/31/33/45/52/58).

A digital anorectal examination and standard anoscopy were performed at each study visit; if these or anal cytologic testing identified abnormalities or confirmed HPV-related perianal lesions, participants underwent high-resolution anoscopy [14]. Visible lesions were biopsied and formalin-fixed for histopathologic assessment and HPV genotyping. Lesions were classified as anal condyloma or AIN grade 1, 2, or 3 according to standard histopathologic criteria; no further classification of lesions was undertaken by p16 immunostaining. The final consensus diagnosis was determined by a central pathology panel. Formalin-fixed paraffin-embedded anal biopsy samples were tested for HPV DNA as described previously, with the use of multiplex PCR assays in a thin section adjacent to the section used for histologic diagnosis [15, 16]. Sections for which a specific HPV type was amplified by PCR assay were classified as HPV positive for that type. In instances in which both AIN1 and AIN2/3 lesions may have been observed in different biopsies (collected on the same date), each lesion was considered separately in the analysis, applying consistent criteria for assigning causality (ie, same HPV type detected in the swab specimen and biopsy sample).

### Analysis Populations and Assessments

Of the 602 MSM who participated in the V501-020 trial [10], all 297 assigned to the placebo arm were included in the current analysis. Two analysis populations were studied. The first analysis population was identified from all MSM assigned to the placebo arm who were HPV naive to the relevant type (HNRT; n = 295), defined as being naive to the HPV type under consideration at day 1. For HPV6/11/16/18, HPV-naive status was defined as having a PCR-negative swab and being serology negative (as assessed by the competitive Luminex immunoassay [17–19]) to the relevant HPV types. HPV31/33/45/52/58 serology information was not available for all participants; therefore, HNRT was defined as having a DNA-negative swab to the relevant HPV type at all anatomical sites, including extra-anal sites. Those with missing PCR results from the intra-anal specimen or a missing serology result at day 1 were excluded from the HNRT analyses. The second analysis was identified from MSM within the full analysis set (FAS) population (n = 297), which included all participants assigned to the placebo arm.

An incident-persistent anal infection was defined as the detection of a new HPV type after the day 1 study visit at 2 or more consecutive visits spaced approximately 6 months apart; outcomes associated with incident-persistent infections were evaluated in the HNRT population. A prevalent-persistent anal infection was defined as the persistence of the specific 9vHPV type infection detected on day 1 at 2 or more consecutive visits spaced approximately 6 months apart; outcomes associated with prevalent-persistent infections were evaluated in the FAS population.

Progression from anal HPV infections to same-type HPV-associated anal disease, including condyloma, AIN1, AIN2, and AIN3, was assessed, along with the distribution of 9vHPV types within each biopsied lesion. An HPV infection was considered to have progressed to associated disease if the HPV type in the biopsy sample was the same as the HPV genotype identified in the intra-anal swab specimen. Swab specimens and biopsy samples could be positive for more than 1 HPV type. In cases where multiple 9vHPV types were identified in both the specimen and biopsy sample, causal HPV type could not be assigned. As a result, each HPV type in multi-type infections was considered causal if detected in the corresponding biopsy sample.

### Statistical Analysis

Kaplan–Meier methods were used to estimate the cumulative incidence of progression from incident-persistent anal HPV infection (HNRT population) or prevalent-persistent anal infection (FAS population) to same-type HPV-associated anal disease (anal condyloma/AIN1, AIN2, and AIN3) over 30 months of follow-up. Among MSM with infections that progressed to anal disease, median time in months from incident-persistent or prevalent-persistent HPV infection to associated anal lesions was calculated; MSM with infections that did not progress to same-type HPV-associated anal disease were censored. The incidence of anal condyloma and AIN (AIN1, AIN2, and AIN3) by 9vHPV type, irrespective of baseline HPV infection status, was also assessed.

The association between baseline characteristics and risk of progression from incident-persistent anal infection from any 9vHPV type to same-type HPV-associated anal disease was assessed by logistic regression analysis. Odds ratios and corresponding 95% confidence intervals were estimated, with adjustment for age. All analyses were conducted using SAS version 9.4.

## RESULTS

### Demographic and Behavioral Characteristics (HNRT Population)

Most MSM in the placebo arm of the V501-020 trial were aged 21–27 years (69.5%), and most were enrolled from North America (42.7%), Latin America (22.7%), and Europe (20.7%) (Table 1). Many MSM in this study had 4–5 lifetime male sex partners (46.8%) and 3–5 lifetime partners with insertive (43.1%) and receptive (41.6%) anal intercourse.

**Table 1.** Baseline Demographic and Behavioral Characteristics of the Study (HNRT) Population^a^

	MSM (N = 295) n (%)
Age, y^b^	
16–20	90 (30.5)
21–27	205 (69.5)
Geographic region	
North America	126 (42.7)
Latin America	67 (22.7)
Europe	61 (20.7)
Asia-Pacific	41 (13.9)
Africa	0
Tobacco use on day 1	
Never used	147 (49.8)
Ex-users	29 (9.8)
Current user	119 (40.3)
Age at first intercourse, y^c^	
<15	36 (12.6)
15–19	195 (68.2)
≥20	55 (19.2)
Number of lifetime male sex partners^c^	
0–3	150 (53.2)
4–5	132 (46.8)
Number of lifetime female sex partners^c^	
0–3	76 (98.7)
4–5	1 (1.3)
Number of lifetime partners with insertive anal intercourse^c^	
0	33 (11.7)
1	73 (26.0)
2	54 (19.2)
3–5	121 (43.1)
Number of lifetime partners with receptive anal intercourse^c^	
0	31 (11.0)
1	63 (22.4)
2	70 (24.9)
3–5	117 (41.6)
Number of new male partners in past 6 m^c^	
0	105 (37.2)
1	109 (38.7)
≥2	68 (24.1)
Number of new female partners in past 6 m^c^	
0	72 (93.5)
1	4 (5.2)
≥2	1 (1.3)
Frequency of lifetime condom use^c^	
Always	107 (36.3)
More than half the time	133 (45.1)
Less than half the time	25 (8.5)
Never	18 (6.1)
Frequency of condom use in the past 6 m	
Always	114 (38.6)
More than half the time	68 (23.1)
Less than half the time	24 (28.1)
Never	74 (25.1)
Circumcision	
No	166 (56.3)
Yes	129 (43.7)
Follow-up characteristics	
Mean follow-up, m	28.3^d^
Median follow-up, m	32.7^e^

Abbreviations: FAS, full analysis set; HPV, human papillomavirus; HNRT, HPV naive to relevant type; MSM, men who have sex with men; PCR, polymerase chain reaction.

^a^HNRT population defined as HPV naive at day 1 to the HPV type under consideration. For HPV 6, 11, 16, and 18, HPV naive was defined as having a PCR-negative swab and serology negative to the relevant HPV type. For HPV 31, 33, 45, 52, and 58, HPV naive was defined as having a PCR-negative swab to the relevant HPV type.

^b^The inclusion criterion for age was 16–26 years; however, some men aged 26 years at screening subsequently turned age 27 years by the enrollment visit.

^c^Categories have missing values for MSM.

^d^Mean follow-up in the FAS population was 30.1 months.

^e^Median follow-up in the FAS population was 35.9 months.

### Prevalence of 9vHPV Vaccine Types in Intra-anal Swabs and Lesion Biopsies (Full Analysis Set Population)

On day 1, 9vHPV DNA was detected in intra-anal specimens from 99 (33.3%) MSM (Table 2), with HPV6 (11.5%) and HPV16 (12.5%) the most common types; furthermore, 66 (22.2%) MSM had multiple 9vHPV types detected within the same specimen, with HPV16 (10.1%), HPV6 (8.8%), HPV18 (5.4%), and HPV45 (5.4%) the most common types (Supplementary Table 1).

**Table 2.** Distribution of 9vHPV Types in Anal Swabs and Condyloma/AIN1, AIN2, AIN3, and Pooled AIN2 or AIN3 Biopsies Among MSM (FAS Population^a^)

HPV Type,^b^ n (%)^c^	HPV Infection in the Intra-anal Swab on Day 1 (N = 297)	Anal Condyloma/AIN1 Biopsies (N = 79)	AIN2 Biopsies (N = 49)	AIN3 Biopsies (N = 27)	AIN2 or AIN3^d^ Biopsies (N = 59)
6	34 (11.5)	44 (55.7)	13 (26.5)	8 (29.6)	18 (30.5)
11	16 (5.4)	20 (25.3)	6 (12.2)	5 (18.5)	9 (15.3)
16	37 (12.5)	7 (8.9)	8 (16.3)	8 (29.6)	13 (22.0)
18	19 (6.4)	6 (7.6)	5 (10.2)	5 (18.5)	7 (11.9)
31	15 (5.1)	2 (2.5)	1 (2.0)	7 (25.9)	7 (11.9)
33	10 (3.4)	0	1 (2.0)	7 (25.9)	2 (3.4)
45	20 (6.7)	5 (6.3)	2 (4.1)	1 (3.7)	2 (3.4)
52	17 (5.7)	2 (2.5)	3 (6.1)	0	6 (10.2)
58	13 (4.4)	5 (6.3)	2 (4.1)	1 (3.7)	3 (5.1)
6 or 11 only^e^	10 (3.4)	28 (35.4)	5 (10.2)	3 (11.1)	8 (13.6)
16 or 18 only^f^	10 (3.4)	1 (1.3)	3 (6.1)	3 (11.1)	5 (8.5)
6/11/16/18	77 (25.9)	62 (78.5)	29 (59.2)	19 (70.4)	39 (66.1)
31/33/45/52/58	52 (17.5)	12 (15.2)	8 (16.3)	10 (37.0)	17 (28.8)
6/11/16/18/31/33/45/52/58	99 (33.3)	63 (79.8)	33 (67.4)	24 (88.9)	47 (79.7)
Unknown^g^	0	16 (20.3)	14 (28.6)	2 (7.4)	15 (25.4)

Abbreviations: 9vHPV, 9-valent HPV; AIN, anal intraepithelial neoplasia; FAS, full analysis set; HPV, human papillomavirus; MSM, men who have sex with men.

^a^The FAS population was defined as all participants assigned to the placebo arm.

^b^With the exception of HPV6 or HPV11 only and HPV16 or HPV18 only, participants could be infected with multiple HPV types, and biopsies could be positive for multiple HPV types. A causal HPV type was not assigned in these cases in which multiple HPV types were present.

^c^“n” indicates the number of participants with a specific lesion.

^d^MSM had AIN2, AIN3, or both, but they are counted only once.

^e^Infection with HPV6 only or HPV11 only.

^f^Infection with HPV16 only or HPV18 only.

^g^Not related to any of the 9vHPV types.

The predominant 9vHPV types detected in anal condyloma/AIN1 swabs were HPV6 (55.7%) and HPV11 (25.3%); only HPV6 or HPV11 was detected in 35.4% of lesions, with no other 9vHPV type present (Table 2). Individual high-risk 9vHPV types (16/18/31/33/45/52/58) were detected in 0%–9% of condyloma/AIN1 cases, with 1.3% of cases having HPV16 or HPV18 only and 15.2% having any of the other high-risk 9vHPV types (31/33/45/52/58) in the absence of low-risk types (HPV6/11). The most common 9vHPV genotypes that were detected in AIN2 and AIN3 lesions were HPV6 (26.5% and 29.6%, respectively), HPV16 (16.3% and 29.6%, respectively), and HPV11 (12.2% and 18.5%, respectively) (Table 2); however, the proportions of cases of HPV6 or HPV11 infection alone detected in AIN2 and AIN3 lesions were low (10.2% and 11.1%, respectively). HPV18/31/33 were also frequently detected in AIN3 lesions. Similar findings were observed for pooled AIN2 or AIN3 data.

Multiple 9vHPV types within the same lesion were identified in 34.2% of MSM with anal condyloma/AIN1, 22.4% of those with AIN2, and 48.1% of those with AIN3 (Supplementary Table 1). The most common 9vHPV genotypes occurring in lesions with multiple HPV types were HPV6 (26.6%) and HPV11 (10.1%) in MSM with anal condyloma/AIN1 and HPV6 (11.9%), HPV11 (11.9%), HPV16 (11.9%), HPV18 (8.5%), and HPV31 (10.2%) in the pooled population with AIN2 or AIN3.

### Incident-persistent and Prevalent-persistent Intra-anal 9vHPV Infections and Progression to AIN1/AIN2/AIN3

Overall, 30.2% of MSM had incident-persistent intra-anal 9vHPV infections, which included 13.3% with HPV6, 13.2% with HPV16/18, 6.9% with HPV11, and 14.6% with HPV31/33/45/52/58 (Table 3). Of MSM with an incident-persistent intra-anal 9vHPV infection, 43.8% had infections that progressed to anal condyloma/AIN1 (HPV6, 63.0%; HPV11, 85.7%; HPV16/18, 16.3%) as determined by biopsy, and 33.7% had infections that progressed to AIN2 or AIN3 (HPV6, 25.9%; HPV11, 14.3%; HPV16/18, 32.4%) as determined by biopsy.

**Table 3.** Proportion of MSM With Incident-Persistent Intra-anal 9vHPV Infection (HNRT Population^a^) and Prevalent-Persistent Intra-anal 9vHPV Infection (FAS Population^b^)

	Incident-Persistent HPV Infection^c^			Prevalent-Persistent HPV Infection^d^		
HPV Type, %	Proportion with infection (n^e^/N^f^)	Progression to anal condyloma/AIN1 *(n*^g^/N^h^)	Progression to AIN2 or AIN3 (n^g^/N^h^)	Proportion with infection (n^i^/N^j^)	Progression to anal condyloma/AIN1 (n^k^/N^l^)	Progression to AIN2 or AIN3 (n^k^/N^l^)
HPV6	13.3 (27/203)	63.0 (17/27)	25.9 (7/27)	8.1 (24/297)	66.7 (16/24)	20.8 (5/24)
HPV11	6.9 (14/203)	85.7 (12/14)	14.3 (2/14)	3.0 (9/297)	66.7 (6/9)	44.4 (4/9)
HPV16/18	13.2 (37/281)	16.2 (6/37)	32.4 (12/37)	11.4 (34/297)	23.5 (8/34)	26.5 (9/34)
HPV31/33/45/52/58	14.6 (43/295)	16.3 (7/43)	23.3 (10/43)	9.8 (29/297)	31.0 (9/29)	34.5 (10/29)
Any 9vHPV^m^	30.2 (89/295)	43.8 (39/89)	33.7 (30/89)	22.6 (67/297)	53.7 (36/67)	41.8 (28/67)

Abbreviations: 9vHPV, 9-valent HPV; AIN, anal intraepithelial neoplasia; FAS, full analysis set; HNRT, HPV naive to relevant type; HPV, human papillomavirus; MSM, men who have sex with men; PCR, polymerase chain reaction.

^a^HNRT population defined as HPV naive at day 1 to the HPV type under consideration. For HPV6, HPV11, HPV16, and HPV18, HPV naive was defined as having a negative swab (ie, PCR negative) and serology negative to the relevant HPV type. For HPV31, HPV33, HPV45, HPV52, and HPV58, HPV naive was defined as having a negative swab to the relevant HPV type.

^b^The FAS population was defined as all participants assigned to the placebo arm.

^c^An incident-persistent infection was defined as the detection of a new HPV type after the day 1 study visit at 2 or more consecutive visits spaced approximately 6 months apart (± 1-month window for each visit).

^d^A prevalent-persistent infection was defined as the persistence of the specific 9vHPV type prevalent infection at 2 or more consecutive visits spaced approximately 6 months apart (± 1-month window for each visit).

^e^Number of MSM from the HNRT population with incident-persistent infection for a given 9vHPV infection type at the intra-anal site.

^f^Number of MSM at risk for a given 9vHPV infection type at the intra-anal site in the HNRT population.

^g^Number of MSM with a given 9vHPV type of incident-persistent infection that progressed to specified anal lesion during follow-up.

^h^Number of MSM with a given 9vHPV type of incident-persistent infection.

^i^Number of MSM from the FAS population with prevalent-persistent infection for a given 9vHPV infection type at the intra-anal site.

^j^Number of MSM at risk for a given 9vHPV infection type at the intra-anal site in the FAS population.

^k^Number of MSM with a given 9vHPV type of prevalent-persistent infection that progressed to specified anal lesion during follow-up.

^l^Number of MSM with a given 9vHPV type of prevalent-persistent infection.

^m^MSM with more than 1 9vHPV type are counted only once.

Similarly, 22.6% of MSM had prevalent-persistent intra-anal 9vHPV infection, including 8.1% with HPV6, 11.4% with HPV16/18, 3.0% with HPV11, and 9.8% with HPV31/33/45/52/58 (Table 3). Of MSM with a prevalent-persistent 9vHPV infection at the intra-anal site, 53.7% had infections that progressed to anal condyloma/AIN1 (HPV6, 66.7%; HPV11, 66.7%; HPV16/18, 23.5%) and 41.8% had infections that progressed to AIN2 or AIN3 (HPV6, 20.8%; HPV11, 44.4%; HPV16/18, 26.5%).

### Time to the Development of Associated Anal Disease

The median time from detection of anal 9vHPV infection to development of anal condyloma/AIN1 over 30 months’ follow-up was longer in MSM with incident-persistent infections than in those with prevalent-persistent infections (6.33 vs 2.33 months, respectively) (Table 4). A similar trend was also observed for progression from anal 9vHPV infections to pooled AIN2 or AIN3; the median time to progression was longer in MSM with incident-persistent infections (AIN2 data only) than in those with prevalent-persistent infections (7.43 vs 5.27 months, respectively) (Table 4). Median times to progression from an incident-persistent anal HPV6 infection to anal condyloma/AIN1 and progression from an incident-persistent anal HPV16/18 infection to pooled AIN2 or AIN3 were similar (both 7.4 months). Median times of <2 months were observed for progression from prevalent-persistent anal HPV6 or HPV11 infection to anal condyloma/AIN1 and for progression from incident-persistent or prevalent-persistent HPV11 anal infection to AIN2 or AIN3.

**Table 4.** Median Time in Months From Incident-Persistent or Prevalent Persistent HPV Infection to Associated Anal Lesions Among MSM (FAS Population^a^)

HPV Type	Anal Condyloma/AIN1		AIN2 or AIN3	
	Incident-persistent infection	Prevalent-persistent infection	Incident-persistent infection	Prevalent-persistent infection
HPV6	7.40	1.17	11.12	4.05
HPV11	1.95	2.30	1.77	1.88
HPV16/18	7.43	2.40	7.40	4.30
HPV31/33/45/52/58	5.73	12.73	12.60	7.6
9vHPV	6.33	2.33	7.43	5.27

Abbreviations: 9vHPV, 9-valent HPV; AIN, anal intraepithelial neoplasia; FAS, full analysis set; HPV, human papillomavirus; MSM, men who have sex with men.

^a^The FAS population was defined as all participants assigned to the placebo arm.

### Cumulative Incidence of Anal Condyloma/AIN1

**Figure 1.**
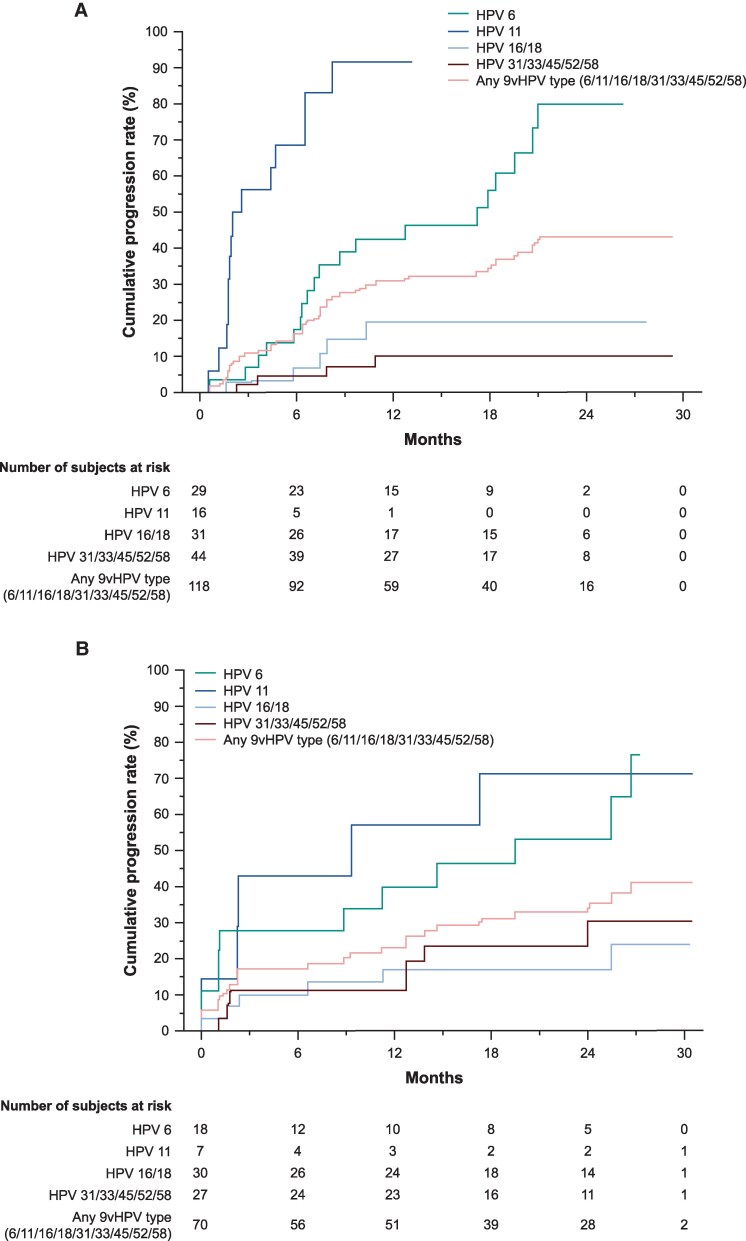
Kaplan–Meier estimates for the cumulative incidence of progression from incident-persistent (*A*) or prevalent-persistent (*B*) anal HPV infections^a^ with 9vHPV types (6/11/16/18/31/33/45/52/58) based on swab sampling, to anal condyloma/AIN1 among MSM (HNRT population^b^) associated with the same HPV type. 9vHPV, 9-valent HPV; AIN, anal intraepithelial neoplasia; HNRT, HPV naive to relevant type; HPV, human papillomavirus; MSM, men who have sex with men; PCR, polymerase chain reaction. ^a^Infection with ≥1 9vHPV type. ^b^HNRT population defined as HPV naive at day 1 to the HPV type under consideration. For HPV6, HPV11, HPV16, and HPV18, HPV naive was defined as having a negative swab (ie, PCR negative) and serology negative to the relevant HPV type. For HPV31, HPV33, HPV45, HPV52, and HPV58, HPV naive was defined as having a negative swab to the relevant HPV type.

The cumulative incidence of anal condyloma/AIN1 among MSM with incident-persistent 9vHPV infections over 30 months’ follow-up was high (43.1%) and was driven by progression from HPV6/11 infections (cumulative incidence, 79.9% and 91.7%, respectively) (Figure 1*A*). Among MSM with incident-persistent HPV16/18 infections that progressed to anal condyloma/AIN1, HPV6/11 co-infection was absent in 1 individual and present in 4 individuals. The cumulative incidence of anal condyloma/AIN1 among MSM with prevalent-persistent 9vHPV infections was 41.2% (Figure 1*B*) and was also driven by progression of HPV6/11 infections (cumulative incidence, 53.2% and 71.4%, respectively). Among MSM with prevalent-persistent HPV16/18 infections that progressed to anal condyloma/AIN1, HPV6/11 co-infection was absent in 3 individuals and present in 5 individuals.

### Cumulative Incidence of Pooled AIN2 or AIN3

**Figure 2.**
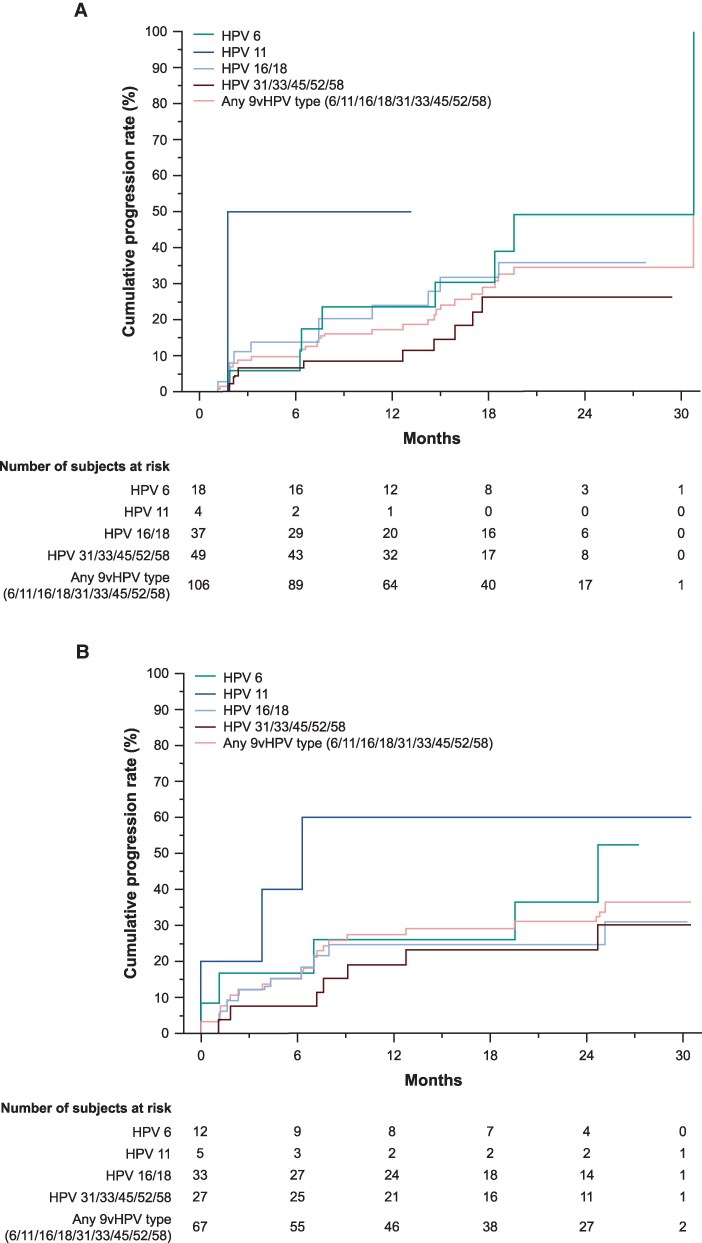
Kaplan–Meier estimates for the cumulative incidence of progression from incident-persistent (*A*) or prevalent-persistent (*B*) anal HPV infections with 9vHPV types (6/11/16/18/31/33/45/52/58) based on swab sampling, to AIN2 or AIN3 among MSM (HNRT population^a^) associated with the same HPV type. 9vHPV, 9-valent HPV; AIN, anal intraepithelial neoplasia; HNRT, HPV naive to relevant type; HPV, human papillomavirus; MSM, men who have sex with men; PCR, polymerase chain reaction. ^a^HNRT population defined as HPV naive at day 1 to the HPV type under consideration. For HPV6, HPV11, HPV16, and HPV18, HPV naive was defined as having a negative swab (ie, PCR negative) and serology negative to the relevant HPV type. For HPV31, HPV33, HPV45, HPV52, and HPV58, HPV naive was defined as having a negative swab to the relevant HPV type.

The cumulative incidence of pooled AIN2 or AIN3 among MSM with incident-persistent infection over 30 months’ follow-up was 34.4% and was driven by progression from HPV6 and HPV16/18 infections (49.1% and 35.9%, respectively) (Figure 2*A*). Among MSM with incident-persistent HPV6/11 infection that progressed to AIN2 or AIN3, co-infection with other 9vHPV types was absent in 9 individuals, and HPV16/18 co-infection was present in 4 individuals. The cumulative incidence of pooled AIN2 or AIN3 in MSM with prevalent-persistent infection was 36.5% (Figure 2*B*), driven by HPV11 and HPV16/18 infections (60.0% and 30.9%, respectively). Among MSM with prevalent-persistent HPV6/11 infections that progressed to AIN2 or AIN3, co-infection with other 9vHPV types was absent in 10 individuals, with no HPV16/18 co-infections found.

### Incidence of Anal Condyloma/AIN1, AIN2, and AIN3 by Lesion 9vHPV Type

The incidence of 9vHPV-associated anal condyloma/AIN1 was 5.15 per 100 person-years (Supplementary Table 2); the highest incidences of individual 9vHPV genotypes associated with anal condyloma/AIN1 were HPV6 and HPV11 (3.96 and 2.52 per 100 person-years, respectively). In addition, the incidence of anal condyloma/AIN1 in MSM with HPV6 only was also high (3.14 per 100 person-years).

The incidence rates of 9vHPV-associated AIN2 and AIN3 among MSM were 2.82 and 2.08 per 100 person-years, respectively (Supplementary Table 2), with the highest incidences associated with HPV6 and HPV16 in both AIN2 (1.24 and 0.91 per 100 person-years, respectively) and AIN3 (0.82 and 0.73 per 100 person-years, respectively). Similar trends were observed for pooled AIN2 or AIN3 data. In MSM with HPV6 only and HPV16 only, the incidence rates of both AIN2 (0.70 and 0.42 per 100 person-years, respectively) and AIN3 (0.47 and 0.21 per 100 person-years, respectively) were low. No cases of anal cancer were reported.

### Baseline Characteristics Associated With Risk of Progression to Associated Anal Disease

Progression from an incident-persistent 9vHPV anal infection to anal condyloma/AIN1 was more likely in MSM who were circumcised than in those who were uncircumcised (adjusted odds ratio, 3.50; 95% confidence interval, 1.34–9.14). Other factors assessed were not associated with risk of progression of incident-persistent 9vHPV infections to same-type HPV-associated anal disease, including age, geographic region, tobacco use, and sexual behaviors (data not shown).

## DISCUSSION

This study assessed the progression of anal HPV infections to same-type HPV-associated anal lesions among unvaccinated HIV-negative MSM aged 21–27 years. Progression from incident-persistent intra-anal 9vHPV infection to anal disease was driven by HPV6, HPV11, or HPV16/18 for MSM with anal condyloma/AIN1 and AIN2/3. Similar trends were observed for progression from prevalent-persistent intra-anal 9vHPV infection to anal disease. Cumulative incidences of anal condyloma/AIN1 and AIN2/3 were high in MSM with an incident-persistent infection or a prevalent-persistent infection. Our findings highlight that a high proportion of unvaccinated, young HIV-negative MSM with intra-anal incident-persistent and prevalent-persistent 9vHPV infection developed same-type HPV-associated anal disease.

To our knowledge, this is the first report describing progression from prevalent-persistent or incident-persistent anal HPV infection to incident anal disease among young HIV-negative MSM aged 21–27 years. Understanding the HPV types associated with lesions and the early stages of the natural history of progression from anal disease in HIV-negative MSM is important because this group represents a substantial proportion of anal HPV-related anal disease cases, including cancer [3, 9]. These data are also important to understanding the risk of anal HPV infection and progression to anal disease in an unvaccinated MSM population.

Among those with prevalent-persistent or incident-persistent anal HPV infections, progression to AIN2 and AIN3 was common during 18 months’ follow-up. HPV6, HPV16/18, and HPV31/33/45/52/58 were each associated with approximately 20% of men developing AIN2. These HPV types/groups had similar incidences of AIN3, with approximately 20% developing AIN3. Similar rates of progression from incident-persistent or prevalent-persistent anal HPV infections to AIN2 or AIN3 were observed. This finding in a cohort of young MSM suggests that the prevalent infection detected at baseline in the study was recently acquired, as the rate of progression of prevalent infections to disease has been shown to be higher than incident infections at other anatomical sites.

The cumulative incidence of HPV6/11-associated condyloma/AIN1 was high, irrespective of whether the infection was detected as prevalent-persistent or incident-persistent. Among those with incident-persistent HPV6 infection, approximately 50% of infections progressed to anal condyloma/AIN1 within 18 months, and 80% within 2 years. As with incident AIN2 or AIN3, those with prevalent-persistent HPV infections and those with incident-persistent HPV infections had little difference in incident condyloma/AIN1.

Our data show that AIN2 or AIN3 lesions develop quickly after HPV infection among MSM who were young and HIV negative. The significance of this finding for future anal cancer risk is unclear. We hypothesize that AIN2 or AIN3 lesions associated with HPV16 or HPV18 are more likely to persist than those associated with other HPV types. However, little is known about the natural history of these lesions in such a young male population and whether these high-grade lesions will eventually progress to cancer if left untreated, or undergo spontaneous regression [20, 21].

This study has several strengths and limitations. A strength was the availability of data from a large MSM population across several geographic regions. Additionally, the study included a sensitive and reproducible HPV genotyping platform using a central laboratory for HPV DNA typing of anal swabs and anal lesions, where lesion grade was confirmed by central pathology review. Furthermore, the young age of MSM enrolled in the study allowed us to examine the natural progression of an HPV infection to same-type HPV-associated anal disease early after infection.

A limitation of this analysis is the lack of data on anal lesions that are driven by non-vaccine-targeted HPV types. Although 9vHPV vaccine types cover most HPV-associated low- and high-grade anal lesions, a small proportion of these lesions are caused by other HPV types. Furthermore, previous evidence indicates that high-grade anal lesions are primarily driven by high-risk HPV types and that single lesions are most likely caused by infection with a single HPV type [22]. As a result, the current analysis cannot exclude the involvement of non-9vHPV types for cases of HPV6/11-associated AIN2/3 without co-infection with other 9vHPV types or HPV16/18-associated anal condyloma/AIN1without HPV6/11 co-infection. Short median times to progression were also reported for some HPV types, which may be due partly to workup of concomitant infections/suspected lesions detected at the same clinic visit. Additionally, restricting analyses to MSM with infections that progressed to anal disease during the observational follow-up period (ie, excluding infections that had not yet progressed by the end of the study) may also have contributed to the short median times reported in Table 4. Despite the availability of a relatively large MSM cohort, the sample size limited our ability to assess potential risk factors for progression to incident HPV-associated disease. For example, being circumcised was associated with a higher risk of anal condyloma/AIN1 but not AIN2 or AIN3. The explanation for this finding is unclear and may be spurious. Last, due to MSM eligibility criteria in the V501-020 trial (aged 16–26 years; 1–5 lifetime sex partners; HIV negative), results of this analysis may not be applicable to MSM who are HIV positive, older than 27 years, or who had more than 5 lifetime sex partners.

In summary, our data show that among unvaccinated MSM who were young (aged 21–27 years), had no more than 5 lifetime sex partners, were HIV negative but positive for 1 or more HPV types at study entry or who acquired a new infection, a high proportion developed anal disease spanning the spectrum from condyloma/AIN1 to AIN3 associated with those types. The highest incidence was seen for condyloma/AIN1 in conjunction with HPV6, but high rates of AIN2 or AIN3 were also found together with a broad range of HPV types, including HPV6 and HPV16. Given that an incident anal HPV infection results in a high likelihood of progression to anal disease among young HIV-negative MSM, our data strongly support vaccination to prevent anal HPV infection and associated anal disease in this population.

## Supplementary Material

ciaf584_Supplementary_Data

## Supplementary Data

Supplementary materials are available at *Clinical Infectious Diseases* online. Consisting of data provided by the authors to benefit the reader, the posted materials are not copyedited and are the sole responsibility of the authors, so questions or comments should be addressed to the corresponding author.
