# Supplementary material for: Anal Human Papillomavirus Infection Progression to Disease Among Men Who Have Sex With Men
Source: Clin Infect Dis. 2025 Oct 29;82(3):e616–25. doi: 10.1093/cid/ciaf584 (PMC13016981; doi:10.1093/cid/ciaf584)
Supplement: ciaf584_Supplementary_Data [file ciaf584_supplementary_data.docx]

# Supplemental Material*

Palefsky JM, Giuliano AR, Goldstone SE, et al. Anal human papillomavirus infection progression to disease among men who have sex with men. Ann Intern Med. X March 2025. [Epub ahead of print]. doi:XX.XXXX/XXX-XXXX

**Table of Contents**

[**SUPPLEMENTAL TABLES2**](#SUPPLEMENTALTABLES)

[Supplementary Table 12](#SupplementTable1)

[Supplementary Table 24](#SupplementTable2)

*This supplementary material was provided by the authors to give readers further details on their article.

The material was not copyedited.

**SUPPLEMENTAL TABLES**

## **Supplementary Table S1.** Distribution of 9vHPV Types in Anal Condyloma/AIN1, AIN2, AIN3, and Pooled AIN2 or AIN3 Biopsies Among MSM Infected With Multiple 9vHPV Types Only (FAS population^a^)

| HPV type, n (%)^b^ | HPV infection in the intra-anal swab on day 1 (*N* = 297) | Anal condyloma/AIN1  biopsies (*N* = 79) | AIN2  (*N* = 49)  biopsies | AIN3 (*N* = 27)  biopsies | AIN2 or AIN3^c^ (*N* = 59)  biopsies |
| --- | --- | --- | --- | --- | --- |
| 6 | 26 (8.8) | 21 (26.6) | 3 (6.1) | 5 (18.5) | 7 (11.9) |
| 11 | 14 (4.7) | 8 (10.1) | 3 (6.1) | 5 (18.5) | 7 (11.9) |
| 16 | 30 (10.1) | 6 (7.6) | 4 (8.2) | 5 (18.5) | 7 (11.9) |
| 18 | 16 (5.4) | 6 (7.6) | 3 (6.1) | 4 (14.8) | 5 (8.5) |
| 31 | 14 (4.7) | 2 (2.5) | 1 (2.0) | 5 (18.5) | 6 (10.2) |
| 33 | 10 (3.4) | 0 | 1 (2.0) | 1 (3.7) | 2 (3.4) |
| 45 | 16 (5.4) | 5 (6.3) | 1 (2.0) | 0 | 1 (1.7) |
| 52 | 11 (3.7) | 2 (2.5) | 1 (2.0) | 1 (3.7) | 2 (3.4) |
| 58 | 11 (3.7) | 5 (6.3) | 2 (4.1) | 1 (3.7) | 3 (5.1) |
| 6/11/16/18 | 57 (19.2) | 26 (32.9) | 10 (20.4) | 12 (44.4) | 18 (30.5) |
| 31/33/45/52/58 | 39 (13.1) | 12 (15.2) | 5 (10.2) | 6 (22.2) | 11 (18.6) |
| 6/11/16/18/31/33/45/52/58 | 66 (22.2) | 27 (34.2) | 11 (22.4) | 13 (48.1) | 20 (33.9) |

9vHPV = 9-valent HPV; AIN = anal intraepithelial neoplasia; FAS = full analysis set; MSM = men who have sex with men.

^a^ The FAS population was defined as all participants allocated to the placebo arm.

^b^ “n” indicates the number of participants with a specific lesion.

^c^ MSM had AIN2, AIN3, or both, but are counted only once.

## **Supplementary Table S2.** Incidence (per 100 person-years) of Anal Condyloma/AIN1, AIN2, AIN3, and Pooled AIN2 or AIN3 by Lesion 9vHPV Type^a^ Among MSM, Irrespective of Baseline HPV Infection Status

| HPV type in lesion | Anal condyloma/AIN1 (*N* = 34) | | | AIN2 (*N* = 19) | | | AIN3 (*N* = 14) | | | AIN2 or AIN3^b^ (*N* = 27) | | |
| --- | --- | --- | --- | --- | --- | --- | --- | --- | --- | --- | --- | --- |
|  | *n* | Person-years | Incidence (95% CI) | *N* | Person-years | Incidence (95% CI) | *N* | Person-years | Incidence (95% CI) | *N* | Person-years | Incidence (95% CI) |
| 6 | 19 | 479.5 | 3.96 (2.40 to 6.12) | 6 | 485.7 | 1.24 (0.45 to 2.67) | 4 | 486.8 | 0.82 (0.22 to 2.09) | 8 | 485.2 | 1.65  (0.71 to 3.22) |
| 11 | 12 | 476.7 | 2.52 (1.31 to 4.36) | 3 | 485.9 | 0.62 (0.13 to 1.79) | 2 | 485.9 | 0.41 (0.05 to 1.48) | 3 | 485.9 | 0.62  (0.13 to 1.79) |
| 16 | 1 | 549.9 | 0.18 (0.00 to 1.01) | 5 | 549.0 | 0.91 (0.30 to 2.11) | 4 | 549.3 | 0.73 (0.20 to 1.85) | 7 | 548.1 | 1.28  (0.51 to 2.61) |
| 18 | 4 | 606.7 | 0.66 (0.18 to 1.68) | 3 | 607.5 | 0.49 (0.10 to 1.44) | 2 | 608.8 | 0.33 (0.04 to 1.18) | 4 | 606.0 | 0.66  (0.18 to 1.68) |
| 31 | 0 | - | - | 0 | - | - | 3 | 633.8 | 0.47 (0.10 to 1.38) | 3 | 633.8 | 0.47  (0.10 to 1.38) |
| 33 | 0 | - | - | 1 | 661.5 | 0.15 (0.00 to 0.84) | 0 | - | - | 1 | 661.5 | 0.15  (0.00 to 0.84) |
| 45 | 3 | 631.4 | 0.48 (0.10 to 1.38) | 1 | 632.7 | 0.16 (0.00 to 0.88) | 0 | - | - | 1 | 632.7 | 0.16  (0.00 to 0.88) |
| 52 | 0 | - | - | 2 | 633.6 | 0.32 (0.04 to 1.14) | 2 | 633.0 | 0.32 (0.04 to 1.14) | 4 | 633.0 | 0.63  (0.17 to 1.61) |
| 58 | 1 | 656.5 | 0.15 (0.00 to 0.85) | 0 | - | - | 1 | 656.3 | 0.15 (0.00 to 0.85) | 1 | 656.3 | 0.15  (0.00 to 0.85) |
| Any 9vHPV type^a^ | 34 | 660.5 | 5.15 (3.59 to 7.12) | 19 | 673.3 | 2.82 (1.71 to 4.37) | 14 | 674.6 | 2.08 (1.14 to 3.46) | 27 | 669.6 | 4.03 (2.67 to 5.81) |
| 6 only^c^ | 13 | 414.4 | 3.14 (1.68 to 5.31) | 3 | 426.8 | 0.70 (0.15 to 2.04) | 2 | 426.4 | 0.47 (0.06 to 1.68) | 5 | 426.3 | 1.17 (0.38 to 2.72) |
| 16 only^d^ | 1 | 459.9 | 0.22 (0.01 to 1.21) | 2 | 471.6 | 0.42 (0.05 to 1.52) | 1 | 471.8 | 0.21 (0.01 to 1.18) | 3 | 471.6 | 0.64 (0.13 to 1.85) |
| 6 and 16 only^c^ | 0 | - | - | 1 | 541.6 | 0.18 (0.00 to 1.02) | 1 | 542.6 | 0.18 (0.00 to 1.02) | 1 | 541.6 | 0.18 (0.00 to 1.02) |

9vHPV = 9-valent HPV vaccine; AIN = anal intraepithelial neoplasia; CI = confidence interval; HPV = human papillomavirus; MSM = men who have sex with men.

^a^ 9vHPV types are HPV types targeted by the 9-valent HPV vaccine (HPV6/11/16/18/31/33/45/52/58).

^b^ MSM had AIN2, AIN3, or both, but are counted only once.

^c^ No co-infection.

^d^ Infection with HPV6 and HPV16 co-infection alone.
